# Melioidosis DS rapid test: A standardized serological dipstick assay with increased sensitivity and reliability due to multiplex detection

**DOI:** 10.1371/journal.pntd.0008452

**Published:** 2020-07-13

**Authors:** Gabriel E. Wagner, Esther Föderl-Höbenreich, Karoline Assig, Michaela Lipp, Andreas Berner, Christian Kohler, Sabine Lichtenegger, Julia Stiehler, Wisansanee Karoonboonyanan, Nida Thanapattarapairoj, Chidchanok Promkong, Sirikamon Koosakulnirand, Panjaporn Chaichana, Ralf Ehricht, Anne-Marie Gad, Hans H. Söffing, Susanna J. Dunachie, Narisara Chantratita, Ivo Steinmetz

**Affiliations:** 1 Institute of Hygiene, Microbiology and Environmental Medicine, Medical University of Graz, Graz, Austria; 2 Friedrich Loeffler Institute for Medical Microbiology, Greifswald, Germany; 3 Medical Technology Department, Khon Kaen Hospital, Khon Kaen, Thailand; 4 Medical Technology Department, Udon Thani Hospital, Udon Thani, Thailand; 5 Department of Medical Laboratory, Nakhon Phanom Hospital, Nakhon Phanom, Thailand; 6 Department of Microbiology and Immunology, Faculty of Tropical Medicine, Mahidol University, Bangkok, Thailand; 7 Mahidol-Oxford Tropical Medicine Research Unit, Faculty of Tropical Medicine, Mahidol University, Bangkok, Thailand; 8 Leibniz Institute of Photonic Technology (IPHT), Jena, Germany; 9 InfectoGnostics Research Campus, Jena, Germany; 10 Institute of Physical Chemistry, Friedrich Schiller University Jena, Jena, Germany; 11 Senova Gesellschaft für Biowissenschaft und Technik mbH, Weimar, Germany; 12 Centre for Tropical Medicine and Global Health, University of Oxford, Oxford, United Kingdom; University of Texas Medical Branch, UNITED STATES

## Abstract

**Background:**

Melioidosis, caused by *Burkholderia pseudomallei*, is a severe infectious disease with high mortality rates, but is under-recognized worldwide. In endemic areas, there is a great need for simple, low-cost and rapid diagnostic tools. In a previous study we showed, that a protein multiplex array with 20 *B*. *pseudomallei*-specific antigens detects antibodies in melioidosis patients with high sensitivity and specificity. In a subsequent study the high potential of anti-*B*. *pseudomallei* antibody detection was confirmed using a rapid Hcp1 single protein-based assay. Our protein array also showed that the antibody profile varies between patients, possibly due to a combination of host factors but also antigen variations in the infecting *B*. *pseudomallei* strains. The aim of this study was to develop a rapid test, combining Hcp1 and the best performing antigens BPSL2096, BPSL2697 and BPSS0477 from our previous study, to take advantage of simultaneous antibody detection.

**Methods and principal findings:**

The 4-plex dipstick was validated with sera from 75 patients on admission plus control groups, achieving 92% sensitivity and 97–100% specificity. We then re-evaluated melioidosis sera with the 4-plex assay that were previously misclassified by the monoplex Hcp1 rapid test. 12 out of 55 (21.8%) false-negative samples were positive in our new dipstick assay. Among those, 4 sera (7.3%) were Hcp1 positive, whereas 8 (14.5%) sera remained Hcp1 negative but gave a positive reaction with our additional antigens.

**Conclusions:**

Our dipstick rapid test represents an inexpensive, standardized and simple diagnostic tool with an improved serodiagnostic performance due to multiplex detection. Each additional band on the test strip makes a false-positive result more unlikely, contributing to its reliability. Future prospective studies will seek to validate the gain in sensitivity and specificity of our multiplex rapid test approach in different melioidosis patient cohorts.

## Author summary

The Gram-negative environmental pathogen *Burkholderia pseudomallei*, causes the severe disease melioidosis. It is highly endemic in southeast Asia and northern Australia, but recent studies suggest that it is also present in many other parts of the world where it is severely underreported. The latter results from the extremely variable and non-specific clinical manifestations of the disease, lack of clinical recognition, and the global scarcity of good quality laboratories to allow diagnosis from microbiological culture. This is even more unfortunate, as early diagnosis of the disease is indispensable for an effective therapy, since *B*. *pseudomallei* is intrinsically resistant to many antibiotics used for empirical treatment in endemic areas. Therefore, the development of new, standardized and sensitive tools is of high importance for both diagnostics and epidemiology. We focused on the development of a dipstick assay, which is based on the detection of serum antibodies against four *B*. *pseudomallei* specific protein antigens. Here we present a cost effective, simple and rapid melioidosis assay with improved sensitivity that does not depend on sophisticated laboratory equipment and therefore addresses most of the before mentioned obstacles and is easy to manufacture in large scales.

## Introduction

Melioidosis is an infection caused by the Gram-negative bacterium *Burkholderia pseudomallei*, which can usually be found in soil and surface water. A recent study suggests that environmental conditions in large parts of the world might be well-suited for *B*. *pseudomallei* [1]. Up to now, the pathogen has been reported in endemic regions like southeast Asia and northern Australia, and also less frequently in the Middle East, Africa and Central/South America [1–7]. In addition, a more globalized life style leads to an increased number of melioidosis cases in travelers from all over the world [8–11]. It is estimated that there are about 165,000 melioidosis cases per year worldwide, of which 54% of the patients die [1]. Birnie and colleagues suggested that based on data from 2015, melioidosis should be considered a major neglected tropical disease, as its burden is higher than that of many other tropical diseases [12].

Clinical manifestations of melioidosis vary widely, with pneumonia being the most common localized form [13, 14]. Therefore, the diagnosis of melioidosis is challenging, which is mainly attributed to the non-specific symptoms and the lack of sensitivity and specificity of the commonly used diagnostic tools [15–18]. This is compounded by the lack of awareness and dearth of microbiological capacity in many (predicted) endemic areas [18, 19]. This is especially concerning because diabetes is a major risk factor for melioidosis [18, 20]. With four out of five worldwide diabetes patients now living in low- or middle-income countries and the prevalence of diabetes accelerating in these areas [21, 22], increases in melioidosis incidence is a highly plausible scenario.

The diagnostic gold standard, the cultural detection of *B*. *pseudomallei*, lacks sensitivity and is not as fast as needed in many clinical situations [23]. *B*. *pseudomallei* rapid antigen detection assays are likely to have a limited sensitivity, especially for blood and serum samples [24, 25], and at present PCR methods are rather expensive, have not been thoroughly validated [17, 18, 26] and require highly trained staff as well as equipment and reagents which will not be available in the near future in many endemic areas. Current serological methods on the other hand often suffer from a lack of standardization, a low sensitivity and high background seropositivity. This includes the indirect hemagglutination assay (IHA), which is the serological standard test for melioidosis in many endemic regions, whose sensitivity might be as low as 56%, and is commonly positive in the endemic population [27–29]. There is known cross-reactivity of antibody and cellular responses to *B*. *pseudomallei* and environmental *Burkholderia* species of low pathogenicity in both melioidosis patients and endemic controls [30], so the development of a diagnostic test for acute melioidosis with high specificity is highly desirable.

Recently, a new generation of serological assays attracted great attention [31, 32]. A rapid immunochromatography test based on the hemolysin-coregulated protein (Hcp1) with high sensitivity and specificity shows great promise as a point of care (POC) assay, being well standardized and inexpensive [31].

We have previously shown that serologic microarrays for the detection of *B*. *pseudomallei* infections benefit greatly from multiplex detection of complementary antibodies, increasing both sensitivity and specificity if properly optimized [32]. This might be also crucial for POC devices, as our microarray results show that the antibody response against *B*. *pseudomallei* antigens between individuals differs and hence leads to varying antibody patterns [32]. Multiplex assays are also less prone to protein sequence variations of single antigens in different *B*. *pseudomallei* strains, e.g. described by Sahl and colleagues [33], as the other antigens serve as potential serodiagnostic backups. Indeed, an Hcp1 variant associated with low antigenicity has recently been identified [34], corroborating the before mentioned issue of singleplex tests. Furthermore, the simultaneous detection of antibodies against two or more different antigens can increase the reliability of the test, as a false-positive result becomes less likely with every additional positive antigen test line.

In this study we challenged the hypothesis that the beneficial features of multiplex detection can be exploited in a dipstick based rapid test to improve its diagnostic performance. We therefore selected the three best performing serodiagnostic antigens of our previous microarray study, BPSL2096, BPSL2697 and BPSS0477, and the before mentioned Hcp1. Our aim was to develop a standardized, simple and inexpensive assay for quick screenings in clinical settings of resource-limited regions worldwide.

## Materials and methods

### Ethics

Experiments involving human serum were carried out in accordance with The Code of Ethics of the World Medical Association (Declaration of Helsinki) and were approved by the ethics committees of the Faculty of Tropical Medicine, Mahidol University (Submission number TMEC 12–014, approval numbers MUTM 2012–018, MUTM 2014–079, and MUTM 2016–075); of Sappasithiprasong Hospital, Ubon Ratchathani (reference 018/2555); of Udon Thani Hospital (approval number 2/2560); of Khon Kaen Hospital (approval numbers KE600 and 18); of Nakhon Phanom Hospital (approval number NP-EC11-2/2560) and the Oxford Tropical Research Ethics Committee (reference 64–11).

### Serodiagnostic protein antigens

The four serodiagnostic protein antigens BPSL2096, BPSL2697, BPSS0477 and BPSS1498 (see Table 1) were selected based on their sensitivity and specificity published previously by others and us [31, 32].

**Table 1:** Burkholderia pseudomallei protein antigens used in the dipstick assay.

Test band	Locus Tag	Protein Name	Function	Purification^a^
T1	BPSL2096	Hydroperoxide reductase AhpC	detoxification	Strep/SEC
T2	BPSL2697	Molecular chaperone GroEL1	Protein folding and stabilization	Strep/IEX
T3	BPSS0477	Molecular chaperone GroEL2	Protein folding and stabilization	Strep/SEC
T4	BPSS1498	Hemolysin-coregulated protein (Hcp1)	type VI secretion system protein	Strep/SEC

^a^ Purification protocol: Strep = Step-Tactin affinity chromatography, SEC = size exclusion chromatography, IEX = ion exchange chromatography.

PCR primers for the amplification of the BPSS1498 coding sequence were designed with the software OligoPerfect (Thermo Fischer Scientific, USA) and *B*. *pseudomallei* strain K96243 [35] as template. The primer sequence, the restriction enzymes and the expression vector used are shown in S1 Table. *E*. *coli* Top10 (Thermo Fisher, Germany) was used as a cloning host. The sequence of the cloned gene in the obtained plasmid was confirmed by Sanger sequencing. The related information for the other three genes has been published in our previous article [32] and was added to S1 Table.

### Protein expression and purification

Protein expression was carried out in *E*. *coli* Bl21(DE3)pLysS cells (Promega, Germany). Cells were transformed by heat shock and selected with the appropriate antibiotics (chloramphenicol for pLysS plasmid selection and ampicillin or kanamycin for the selection of the expression plasmids, see S1 Table). A 10 ml overnight culture was used to inoculate 1 L of LB medium containing the appropriate antibiotics for protein expression. Cells were grown to an optical density (OD_600_) of 0.6–0.8 at 37°C, protein expression was induced by the addition of 1 mM Isopropyl-β-D-thiogalactopyranosid (IPTG) (Carl Roth, Germany) and carried out at 20°C overnight. Cells were harvested by centrifugation (4,000 x g, 4°C, 30 min), disrupted by sonication (15 min, 0.5 sec on/off cycle, 90% amplitude, Sonoplus HD2070 (Bandelin, Germany)) and cell debris and insoluble compounds removed by centrifuging at 10,000 g at 4°C for an hour.

The recombinant proteins were purified from the cell lysates by gravity flow Strep-Tactin Sepharose (IBA GmbH, Germany) columns according to the manufacturer’s protocol.

Proteins were subjected to a second fast-protein liquid chromatography (FPLC) purification step using either ion exchange or size-exclusion chromatography depending on the protein contaminations. This additional purification step reduces contaminants, which might lead to unspecific antibody binding. Therefore, proteins were dialyzed against FPLC buffer containing 50 mM Na_2_HPOH_4_ pH 8, 150 mM NaCl in the case of BPSS1498 & BPSL2096 or 50 mM Tris pH 7.5, 10 mM MgCl_2_, 0.5 mM KCl, 1 mM EDTA in the case of BPSL2697 & BPSS0477 respectively. BPSL2697 was applied to a HiTrap DEAE FF column (GE Healthcare Bio-Sciences, Germany) and eluted with a linear sodium chloride gradient (0.0–1.0 M). BPSL2096, BPSS0477 and BPSS1498 were applied to a HiLoad 16/600 Superdex 200 pg column (GE Healthcare Bio-Sciences, Germany), equilibrated with the buffer mentioned above and eluted with an isocratic flow. Fractions were analyzed by sodium dodecyl sulfate polyacrylamide gel electrophoresis (SDS-PAGE) and pure fractions where pooled accordingly. Finally, antigens were dialyzed against phosphate buffered saline (PBS) buffer (Carl Roth, Germany) and stored at -20°C until the spraying of the dipsticks. If not stated otherwise all chemicals for buffer preparation were purchased from Carl Roth, Germany in the highest grade available and used as received.

### *E*. *coli* lysate preparation

*E*. *coli* lysate was added to the serum sample to circumvent background signals. The lysate served as a “blocking/capturing agent” for unspecific patient antibodies against *E*. *coli* contaminations (see [36]) resulting from the protein expression. To obtain the *E*. *coli* lysate, 1 L of LB medium was inoculated with 10 ml of an *E*. *coli* Bl21(DE3)pLysS overnight culture and grown to an optical density (OD_600_) of 0.8 at 37°C. Afterwards cells were grown at 20°C overnight, harvested, disrupted and centrifuged as described above for the protein antigen preparation. The lysate was shock frozen in liquid nitrogen and stored at -80°C until use.

### Serum samples

For the primary evaluation of our ***Melioidosis DS*** rapid test we used the same set of human serum samples that were previously characterized (including IHA testing) and utilized for our melioidosis protein array development [32]. Our serum collection consisted of 75 sera from culture-confirmed melioidosis patients upon admission, 100 healthy controls from Thailand from endemic (Ubon Rachathani, n = 75) and non-endemic regions (Bangkok, n = 25) besides 60 anonymized left over sera from routine care of non-endemic German patients (Greifswald) with bacteremia (n = 57) or fungemia (n = 3) [32].

We also tested another 95 samples from Thailand on our dipsticks. These sera were previously classified as false-negative (n = 55) or false-positive (n = 40) on an Hcp1 based lateral flow assay [31]. Twenty-eight of the false-positive sera were drawn from healthy individuals and 12 from patients suffering from other kinds of infections.

### Dipstick spraying

Protein antigens were sprayed on a nitrocellulose membrane (thickness of the membrane: 125–155 μm; pore size: 10μm, capillary flow time: 110–165 [s/4 cm]) at a concentration of 2 mg/ml (in phosphate buffer 10mM, pH 7.3) by a dispenser system (BioDot XYZ-3000 dispensing platform, 1 μL protein solution/cm). Dipstick kits were stored at 4°C.

### Dipstick protocol to investigate the applicable serum dilution range

As high serum concentrations might lead to false-positive and too low concentrations to false-negative results, we first evaluated the applicable serum concentration range of the developed Melioidosis DS test. Therefore, pooled positive sera (M010, M033, M074, M077 (see supplementary S2 Table) and ten additional melioidosis sera, that were not used for further evaluation of the test) or pooled negative sera (two anonymized sera donated from two healthy Austrian individuals) were diluted in the range from 1:6 to 1:10^7^ in the dipstick master mix, consisting of universal casein diluent buffer (UCDB)-Tween running buffer (Senova, Germany), detection antibody (Anti-h IgG gold conjugate, Senova, Germany; diluted 1:50) and the assay control (FITC-BSA gold conjugate, Senova, Germany; diluted 1:50). 50 μl of the solution were transferred into the well of a microtiter plate and a dipstick was put into the solution. Dipsticks were removed after 10 minutes and analyzed after another five minutes of incubation at room temperature by comparing the band intensities to the gold reference card (Senova, Germany).

### Dipstick protocol for detection of *B*. *pseudomallei-*reactive Immunoglobulin G (IgG) antibodies to assess the diagnostic performance of the assay

**Fig 1:**
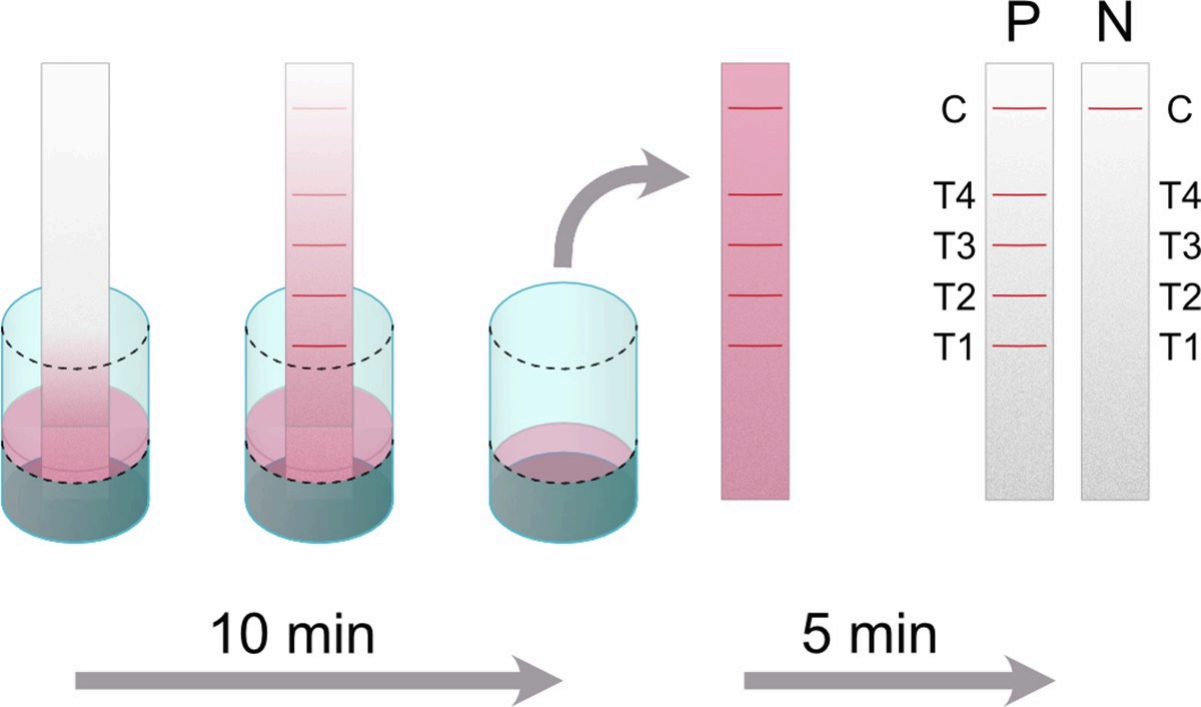
Melioidosis DS assay principle: A dipstick is placed into a well containing patient serum diluted in dipstick master mix (running buffer, detection antibody and control reagent). The mixture flows across the membrane for 10 minutes and patient antibodies labeled with gold-nanoparticle conjugated secondary antibody start to accumulate at the test lines, in case of a specific antibody response. Afterwards the assay is incubated for 5 minutes at room temperature to reduce the background resulting from the flow of the detection antibody. Negative samples only show a single control band, indicating proper assay performance, whereas up to 4 additional bands (T1 –T4) might be visible in case of a positive sample.

Individual serum samples were used to evaluate our ***Melioidosis DS*** assay. Sample names were anonymized and samples from different groups (diseased and controls) were mixed within a single run of 12 samples to avoid bias in the evaluation. For dipstick testing, all of these sera were diluted 1:100 each in dipstick master mix consisting of detection antibody (Anti-h IgG gold conjugate; diluted 1:50), the assay control (FITC-BSA gold conjugate; diluted 1:50) and UCDB-Tween running buffer supplemented with 10% v/v *E*. *coli* lysate as blocking agent. A ***Melioidosis DS*** was dipped into the well of a 96-well plate containing 50 μl of the obtained assay mixture and run for 10 minutes. The dipsticks were removed and evaluated after another 5 minutes of incubation at room temperature (see Fig 1).

All dipsticks were independently evaluated by three individuals to assess the agreement between different evaluators. Of note, as no significant difference between the three different evaluators could be shown in this initial evaluation (see results), ***Melioidosis DS*** assays were evaluated by one experimenter in Thailand, who was trained in the same setting as described above for the primary evaluation of sera.

The assays were analyzed semi-quantitatively by comparing the band intensities to the gold reference card (Senova, Germany). A test was classified as positive if there was at least one visible band present on the dipstick, regardless of the number and intensity of the bands (S2 Table). Nevertheless, the reported semi-quantitative results (S2 Table) for the respective bands will facilitate technical comparison between future dipstick versions aimed at increasing signal intensity and test performance.

### Statistical analysis and data visualization

Statistical analysis has been carried out using IBM SPSS Statistics version 25.0.0.1 (IBM, USA). Reported sensitivities and specificities rely on bacterial culture results, whereas reported confidence intervals are Jeffreys intervals. The Fisher’s exact test was used to test for differences between melioidosis patients and controls for the single antigens. The Cochran’s Q Test for related samples was used to evaluate differences in the diagnostic test performance of different melioidosis tests or due to different evaluators in case of the *Melioidosis DS* assay. Bonferroni correction was used to adjust significance values for multiple tests. A *p* value equally to or smaller than 0.05 was considered significant. Sensitivities and specificities were calculated using the following formulas: sensitivity = ∑ true melioidosis positive tested individuals / ∑ total melioidosis positive individuals; specificity = ∑ true melioidosis negative tested individuals / ∑ total melioidosis negative individuals.

## Results and discussion

### Development of a robust multiplex melioidosis dipstick IgG test (“Melioidosis DS” rapid test) based on four serodiagnostic protein antigens

To combine the advantages of a multiplex approach with a rapid test POC format we decided to develop a 4-plex dipstick (“DS”) assay using the three best performing antigens BPSL2096, BPSL2697, BPSS0477 of our previously published protein microarray [32] and the hemolysin-coregulated protein (Hcp1) BPSS1498. The latter was included as recent studies by Chantratita and colleagues reported a strong serological discrimination power for this antigen [31, 37]. The selected antigens included both *B*. *pseudomallei* GroEL proteins (BPSS0477 and BPSL2697), because mapping the sequence differences on an *E*. *coli* GroEL structure [38] revealed that most of those mismatches belong to surface exposed stretches (see S1 Fig) and hence might very well affect the expression of different epitopes. This is corroborated by our previously published microarray results, where both proteins reacted differently [32]. Protein antigens were sprayed on a nitrocellulose membrane and assembled into the final ***Melioidosis DS*** dipstick test by the addition of an absorption pad.

We then established a simple one-step protocol, in which serum is diluted in the dipstick master mix and the assay is run immediately. Results are obtained in 15 minutes and the setup and assay time are comparable to cassette-based POC tests (Fig 1).

Pooled positive and negative control sera were used to establish the final assay protocol. Additionally, we also evaluated the effects of serum dilution on the signal intensity of the antigen test lines to get an estimate of the range in which our assay distinguishes between sera from diseased individuals and controls. We decided to detect human IgG against *B*. *pseudomallei* antigens as a readout for our assay, as it has been shown to be a valuable diagnostic marker in serological melioidosis assays [31, 39–41]. Although IgM detection has also been applied previously, no clear benefit over IgG based assays has been shown. Some studies even report a lower diagnostic performance for IgM detection [39, 40, 42]. This might be attributed to a strong and fast occurring IgG response, as described by Yi and colleagues in the caprine melioidosis model [43].

**Fig 2:**
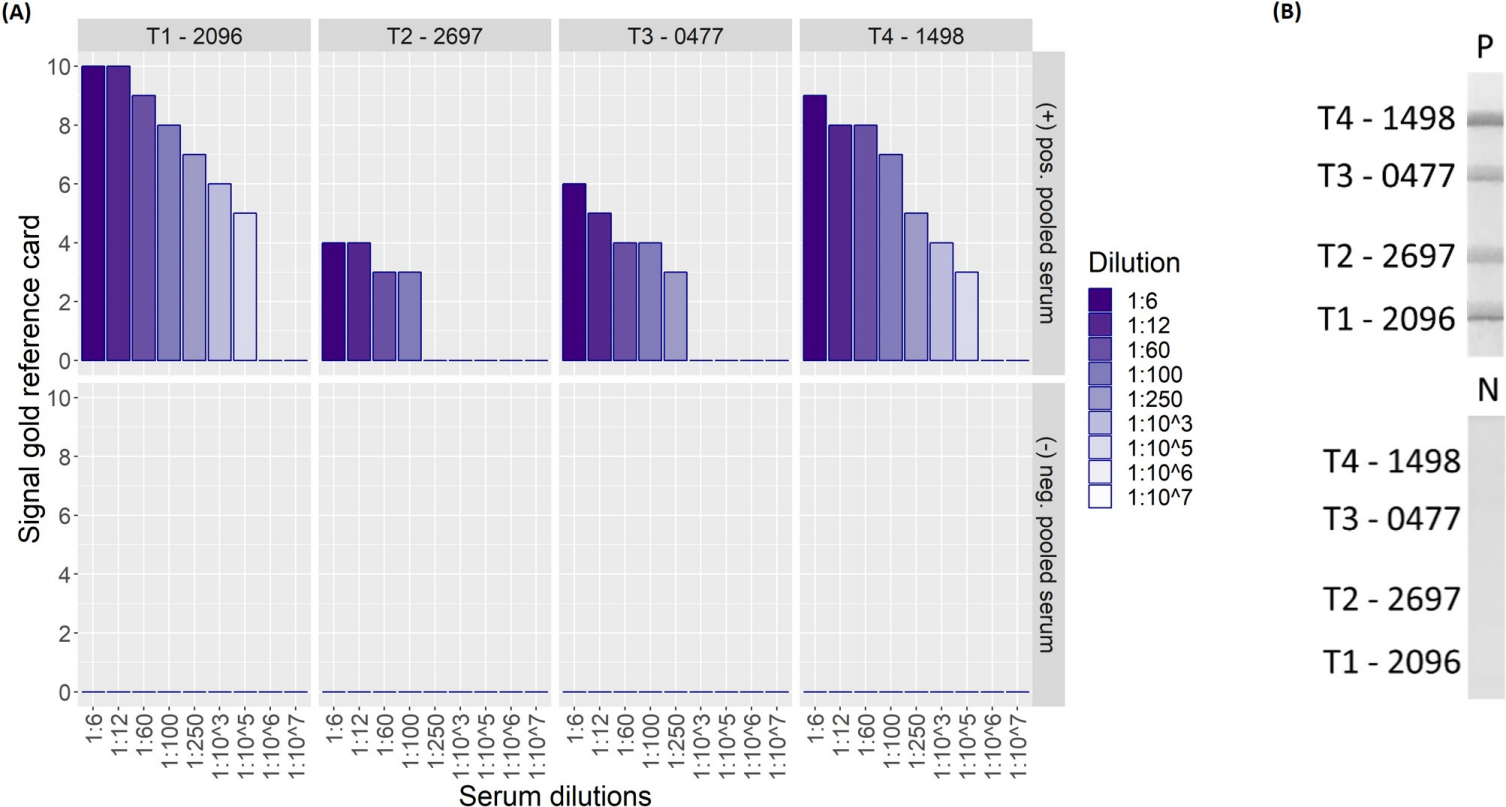
Comparison of Melioidosis DS signal intensities for pooled positive and negative sera over a serum dilution range from 1:6 to 1:10^7^. (A) Signal intensities for all four antigen bands (T1 to T4) are shown for pooled positive and negative serum respectively. The dipstick assay can easily discriminate between these samples up to a dilution of 1:100 for all spotted antigens (T1 to T4). (B) Exemplary dipstick of pooled positive (top) and negative (bottom) serum. A low intensity control line was present on both test strips, but it is not visible on the photographs.

As can be seen in Fig 2 the ***Melioidosis DS*** test shows good performance over a broad range of serum dilutions (1:6–1:100) for all antigens and could clearly discriminate between the pooled positive serum and the control under these conditions. Unsurprisingly, the band intensities were stronger the more serum was being used. In general, broad dilution ranges facilitate assay ease of use, as the system is less dependent on an exact assay setup, which is favorable in point of care settings. Additionally, high serum dilutions might be of particular relevance if there is only access to minimal amounts of blood e.g. after capillary blood sampling, when circumstances do not allow more invasive venous blood sampling. Still it is important to point out, that the data in Fig 2 reflect only the compositions of the pooled sera and hence can only give an estimate about the applicable serum dilution range and its effect on signal intensity.

### Evaluation of the 4-plex *Melioidosis DS* assay shows a promising serodiagnostic performance

**Fig 3:**
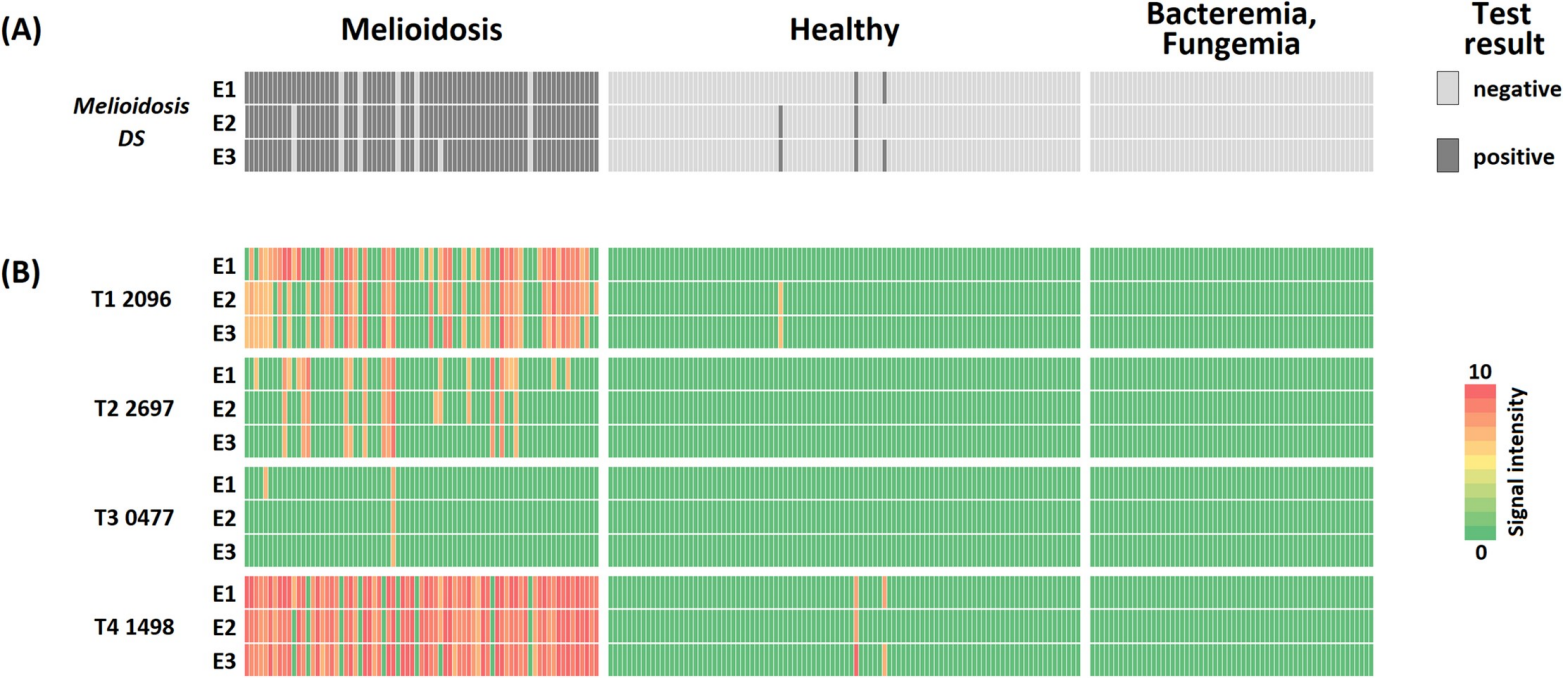
Heat map of melioidosis patient and control **Melioidosis DS** results shown for (A) the overall assay (positive if at least one test band shows a signal) and (B) all four protein antigens separately for three evaluators (E1, E2, E3). The semi-quantitative results for the single antigens were obtained by comparing the dipstick band intensities to the gold reference card. The melioidosis positive sera from were drawn upon admission to the hospital in Thailand (n = 75). The controls consist of healthy individuals from Thailand (n = 100) and German patients suffering from other infections (n = 60). Sera were diluted 1:100 for the Melioidosis DS rapid test.

We then validated our 4-plex ***Melioidosis DS*** assay by using single sera of culture-confirmed melioidosis cases that were drawn on admission alongside respective controls at a serum dilution of 1:100. A heat map of the overall test results for melioidosis sera and controls of three evaluators is shown in Fig 3A.

The Melioidosis DS assay achieved a sensitivity of 92.0% and specificities of 97.0% and 100% for the healthy controls and bacteremia/fungemia controls respectively. The performance was unaffected by excluding GroEL2 (BPSS0477), which did not add significantly to the discrimination between patient sera and controls (see below). We are currently screening for additional serodiagnostic antigens which could potentially replace GroEL2. Of note, all experiments in this study were carried out using a dilution of 1:100, as serum volume was the most limiting factor. The results in Fig 2 indicate that the diagnostic performance of the assay using less dilute serum might lead to even better results, as such assay conditions might increase signals from otherwise false negative samples. As no significant difference between three different evaluators was found (see below), the majority Melioidosis DS result (mode of the three evaluators) is reported. A detailed comparison of the diagnostic parameters including confidence intervals broken down according to evaluator can be found in S4 Table.

We took extra effort to assess test result consistency between evaluators due to the test being read out by eye. However, a comparison between three independent raters showed that they completely agreed for 97.3% of the melioidosis and 98.0% of the control samples (Fig 3A). Statistical analysis revealed no significant difference in the overall assay results (Fig 3A) between the evaluators (*p* = 0.446 for melioidosis and *p* = 1.000 for controls). Of note, Fig 3 not only shows the high consistency of the overall *Melioidosis DS* results (Fig 3A) for all evaluators, but also that very similar band patterns are detected by them (Fig 3B). Sensitivities and specificities according to evaluator split by the antigens are reported in Table 2. On average 1.6–1.8 positive test lines were present in case of melioidosis patients, whereas 0.0 test lines on average were positive for both controls and all evaluators.

**Table 2:** Sensitivity and specificity values reported for single antigens and three different evaluators.

	2096			2697			0477			1498		
Evaluator	E1	E2	E3	E1	E2	E3	E1	E2	E3	E1	E2	E3
**Sensitivity** **(positive)**	60.0%	56.0%	52.0%	28.0%	18.7%	16.0%	2.7%	1.3%	1.3%	89.3%	88.0%	86.7%
**Specificity (healthy)**	100.0%	99.0%	99.0%	100.0%	100.0%	100.0%	100.0%	100.0%	100.0%	98.0%	99.0%	98.0%
**Specificity (bact./fung.)**	100.0%	100.0%	100.0%	100.0%	100.0%	100.0%	100.0%	100.0%	100.0%	100.0%	100.0%	100.0%

Analysis at single antigen level revealed that three out of 75 melioidosis sera would have been wrongly classified as negative if only a singleplex Hcp1 protein dipstick approach had been applied. This translates into a gain of sensitivity from multiplex detection of exactly 4.0% for all evaluators. The Jeffreys interval for sample proportions suggests a benefit in the range from 1.1–10.3% and prospective studies will have to show what can be gained in larger patient cohorts. Of note, the sensitivity of BPSS1498 ranged from 86.7 to 89.3% which is in very good agreement with the previously published value of 88.3% in the rapid immunochromatography HCP1 test by Phokrai and colleagues [31]. These comparable results for BPSS1498 in the lateral flow based POC test [31] and in our dipstick assay further indicate that the results for our assay in this report are indeed a very good estimate for what to expect from a cassette POC version.

A big advantage of multiplex assays is that the reliability of a positive result increases with each additional band. It is less likely that a serum sample contains non-specific antibodies against two or more antigens, hence the chances of obtaining a false-positive result decreases, which contributes to specificity. Therefore, multiplex assays might benefit from each additional antigen. At the moment such an approach is a trade-off between sensitivity and specificity. Still, even for this first generation *Melioidosis DS* test, the consideration of a second positive line leads to a sensitivity of 60.0% already, while ruling out any false-positive result for both control groups (increasing the specificity to 100%). While the drop in sensitivity is not desirable, it means that in our cohort any test with two bands is highly indicative for melioidosis (100%); an information that is unavailable in any singleplex test. Promisingly, if we achieved similar sensitivities per antigen for the four chosen antigens as on the protein microarray [32] a sensitivity of roughly 85% is within reach, even if a positive test result is defined as more than a single positive test line on the dipstick. Therefore, signal amplification techniques, that boost the signal from array-positive but dipstick-negative samples, as well as the identification of novel complementary serological biomarkers for melioidosis are likely to increase its sensitivity in the future.

### Discriminatory power of the single antigens

We re-examined the discriminatory power of the four selected antigens under the test conditions described here by comparing sera from *B*. *pseudomallei* infected individuals and controls (Fisher’s exact test). The antibody response against all antigens except BPSS0477 was significantly different between melioidosis patients and both control groups respectively (healthy individuals and patients with other kind of infections) for all three evaluators (S3 Table). Of note, amongst the chosen microarray antigens, BPSS0477 was already the one with the lowest discriminatory power and weakest average signal intensity [32]. Therefore, it comes with no surprise that it suffers the most from the higher detection limit in the dipstick assays resulting from the readout by eye. Moreover, the dipstick conditions might not be equally suited for all proteins and hence remain a compromise.

### Reevaluation of previously misclassified melioidosis sera verifies the benefit from multiplex detection

Next we applied our test to a collection of 55 sera from culture-confirmed melioidosis cases that were previously misclassified by a Hcp1 single-plex immunochromatographic test [31]. Of note, the original study consisted of 487 melioidose patient sera, of which 430 were positive according to the Hcp1 LFA and two of the 57 false-negative samples were not available anymore [31]. The ***Melioidosis DS*** test results for each serum can be found in S5 Table. 21.8% (12/55) of the previously false-negative samples were correctly identified as melioidosis positive. This is estimated to be a gain of 2.5% (C.I. 1.4–4.2%) in sensitivity using the ***Melioidosis DS*** assay in this cohort. Interestingly, although negative in the other study [31], 7.3% (4/55) of these samples were also BPSS1498 positive on our test, whereas 14.5% (8/55) depend on the other antigens (BPSL2096 and BPSL2697) to obtain a true-positive result. The BPSL2096 test line gave rise to a signal for 5 of these 8 sera and the BPSL2697 test line to 6/8, respectively. The detection of additional BPSS1498 positive samples was unexpected, but can probably be explained by differences in the assay conditions of both tests (different amounts and dilutions of serum used—1:100 vs 1:12-, different running buffers etc.). No difference was observed for the false-positive samples (n = 40) of the previous study [31], which is not surprising as antibodies against BPSS1498 lead to a false positive result in both tests. Evaluating the test for an additional positive band would decrease the number of false-positive tests to 14/40, but also reduce the number of additional true-positive tests to 4/55.

### A comparison of the *Melioidosis DS* to other serological tests illustrates its diagnostic potential

For comparison, the array and IHA results from our previous study [32] can be found in S2 Table and calculated sensitivities and specificities including their confidence intervals for all tests in addition to the here described Melioidosis DS assay, are denoted in S6 Table.

Comparing the three tests revealed a significantly higher sensitivity for the ***Melioidosis DS*** test and the microarray, 92.0% and 86.7% respectively, compared to the 57.3% of the IHA (*p* < 0.001 in each case). No significant difference could be found for the ***Melioidosis DS*** assay and the protein microarray (*p* = 1.000), which is a promising result considering that the ***Melioidosis DS*** test is much cheaper, faster and does not depend on proprietary hardware for analysis. The tests performed equally well on healthy control samples (*p* = 0.867), with specificities of 97.0% (***Melioidosis DS***), 96.0% (IHA) and 97.0% (protein microarray). However, the specificity for bacteremia/fungemia samples is significantly lower for the array compared to the novel dipstick assay (86.7% compared to 100.0%). This might be attributed to the higher detection limit of the dipstick assay due to the readout by eye.

Overall, these first results clearly demonstrate a potential application of the ***Melioidosis DS*** assay in melioidosis serodiagnostics. The 4-plex dipstick assay format allows for a robust selection of up to four antigens to improve its performance over a single antigen based test. At the same time the number of antigens is still low enough to manufacture the test using standard hardware in order to keep the costs of production low. Compared to cassette based POC tests, it offers the advantage that the tests can be run in a 96-well microliter plate, which facilitates handling and parallelization of higher number of samples in a clinical setting for example. The experiment time is comparable to other POC test, but the assay is cheaper as it is just a membrane in principle. Nevertheless, if desired, one could easily obtain a cassette test by the simple addition of a conjugate pad to the dipstick and placing it inside a plastic cartridge.

## Conclusions

In summary, we have shown that multiplexing is a promising approach to enhance sensitivity and test result reliability at the same time, yet still being applicable to an inexpensive, rapid and user-friendly test. Nevertheless, these encouraging results regarding the sensitivity and specificity of our multiplex tool need to be reevaluated in prospective studies with larger cohorts in different parts of the world (including controls for common endemic diseases e.g. leptospirosis) to reveal its true diagnostic performance. The identification of additional antigens might be necessary to further improve sensitivity e.g. by replacing antigens like BPSS0477. In addition, our test offers the chance to analyze whether semi-quantitative (making use of intensity and number of positive test bands) patient antibody profiles are associated with certain clinical conditions and outcome. As such, our assay is the first tool with potential to address such questions for up to four antigens in a rapid test.

## Supporting information

S1 Table Protein antigens used in this study. Locus tag of the protein antigens including the PCR primers, restriction enzymes and the respectively expression plasmids used for cloning.
(XLSX)

S2 Table *Melioidosis DS* assay results. *Melioidosis DS* assay signal intensities for the four spotted antigens tested with melioidosis positive sera and controls. Signal intensities were obtained by comparison of the respective band intensities to the gold reference card (Senova, Germany). *Melioidosis DS* assays were evaluated independently by three individuals. Furthermore, a binary representation (positive—“1”/negative—“0”) is shown for each band besides the overall number of positive bands per assay. Finally, the *Melioidosis DS* assay result is shown, for two conditions: (1) at least one positive band, (2) at least two positive bands. Furthermore, corresponding protein microarray and IHA results for the used sera are included for comparison. Signals of at least two antigens have to be higher than the array threshold of 0.3 for protein microarrays to be considered positive. IHA result lower than 160 were regarded negative.
(XLSX)

S3 Table Results of the Fisher's Exact Test carried out for the four dipstick assay *B*. *pseudomallei* antigens and for all three evaluators. (A) Melioidosis positive samples compared to healthy controls. (B) Melioidosis positive sera compared to bacteremia/fungemia positive samples. p values < 0.05 were considered significantly different between the respective groups. Non-significant differences are indicated by red font color.
(XLSX)

S4 Table *Melioidosis DS* sensitivities and specificities broken down according to evaluator. Melioidosis DS tests were analyzed by three evaluators (E1 –E3). The sample collection consisted of 75 melioidosis positive sera from Ubon Ratchantani, Thailand, 100 healthy controls from Bangkok and Ubon Ratchantani, Thailand and 60 German patient sera suffering from bacteremia or fungemia. Confidence intervals (C.I.) for sensitivities and specificities are Jeffreys intervals.
(XLSX)

S5 Table *Melioidosis DS* assay results for the re-evaluation of the previously misclassified sera. *Melioidosis DS* assay signal intensities for the four spotted antigens tested with Hcp1 singleplex LFA false-negative melioidosis sera and false-positive controls. Signal intensities were obtained by comparison of the respective band intensities to the gold reference card (Senova, Germany). Furthermore, a binary representation (positive—“1”/negative—“0”) is shown for each band besides the overall number of positive bands per assay. Finally, the *Melioidosis DS* assay result is shown for two conditions: (1) at least one positive band, (2) at least two positive bands.
(XLSX)

S6 Table Sensitivities and specificities of the *Melioidosis DS* assay, the indirect hemagglutination assay and the melioidosis protein microarray. The confidence interval specified in parentheses is the Jeffreys interval. IHA is not carried out for routine German bacteremia/fungemia patient samples and is missing therefore.
(XLSX)

S1 Fig Differences in the protein sequence between GroEL1 and GroEL2 mapped on an *E*. *coli* GroEL-GroES complex structure [38]. Mapping these differences (shown in red for one GroEL subunit of the double-heptamer GroEL ring) between GroEL1 and GroEL2 shows that most of those mismatches map to surface exposed residues, which furthermore are not involved in protein-protein interactions. Therefore, the differences in the protein sequence may very well affect the exposed epitopes, which is corroborated by our previous microarray results [32].
(DOCX)
