# Supplementary material for: Melioidosis DS rapid test: A standardized serological dipstick assay with increased sensitivity and reliability due to multiplex detection
Source: PLoS Negl Trop Dis. 2020 Jul 13;14(7):e0008452. doi: 10.1371/journal.pntd.0008452 (PMC7416965; doi:10.1371/journal.pntd.0008452)
Supplement: S1 Fig — Mapping these differences (shown in red for one GroEL subunit of the double-heptamer GroEL ring) between GroEL1 and GroEL2 shows that most of those mismatches map to surface exposed residues, which furthermore are not involved in protein-protein interactions. Therefore, the differences in the protein sequence may very well affect the exposed epitopes, which is corroborated by our previous microarray results [32]. (DOCX) [file pntd.0008452.s007.docx]

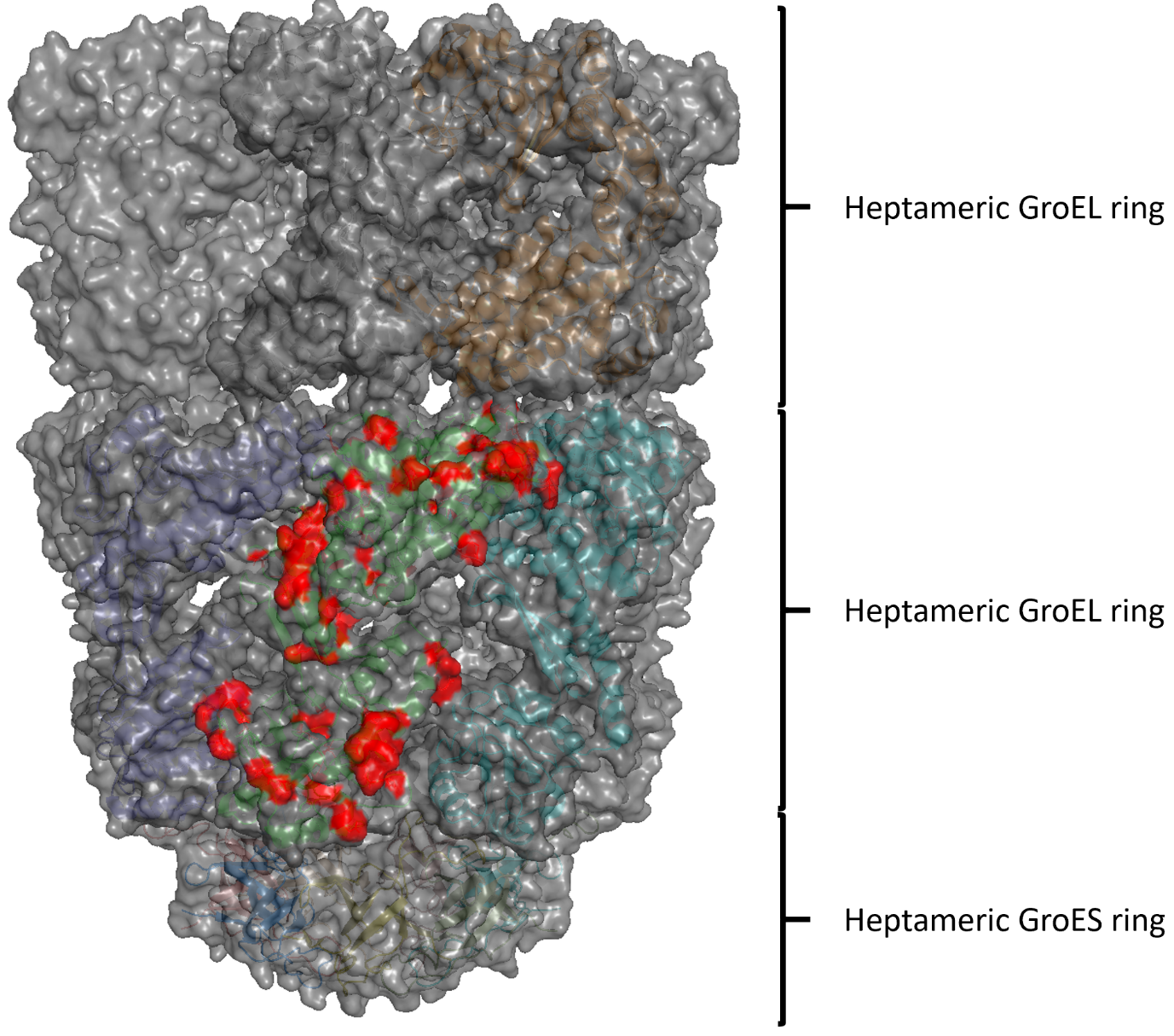


**Figure S 1: Differences in the protein sequence between GroEL1 and GroEL2 mapped on an *E. coli* GroEL-GroES complex structure** [1]. Mapping the differences (shown in red for one GroEL subunit of the double-heptamer GroEL ring) between GroEL1 and GroEL2 shows that most of these mismatches map to surface exposed residues which furthermore are not involved in protein-protein interactions. Therefore, the differences in the protein sequence may very well affect the epitopes which is corroborated by our previous microarray results [2].

1. Ranson NA, Farr GW, Roseman AM, Gowen B, Fenton WA, Horwich AL, et al. ATP-bound states of GroEL captured by cryo-electron microscopy. Cell. 2001;107(7):869-79. Epub 2002/01/10. PubMed PMID: 11779463.

2. Kohler C, Dunachie SJ, Muller E, Kohler A, Jenjaroen K, Teparrukkul P, et al. Rapid and Sensitive Multiplex Detection of Burkholderia pseudomallei-Specific Antibodies in Melioidosis Patients Based on a Protein Microarray Approach. PLoS Negl Trop Dis. 2016;10(7):e0004847. Epub 2016/07/20. doi: 10.1371/journal.pntd.0004847. PubMed PMID: 27427979; PubMed Central PMCID: PMCPMC4948818.
